# St. George's Hospital

**Published:** 1914-05-30

**Authors:** 


### THE DEPARTMENT OF MODERN NURSING.

### St. George's Hospital.

St. George's Hospital was founded in 1733 as an offshoot from the Westminster Hospital. Its position on what eventually became the finest site of modern London was selected in order to procure for the institution the advantages of pure air and country quiet. Now that after the lapse of 150 years the seclusion hoped for in this place of retirement has been considerably modified, amalgamation with the parent hospital on some less cramped site has been decided on. Meantime St. George's presents a good example among the older London hospitals of construction difficulties in the housing of the staff wisely overcome. The average number of occupied beds is 294.

### The First Year of Training.

St. George's has no preliminary school. All the work of fitting the unskilled girl to be useful has therefore to be carried on in the wards. Probationers serve for one month on trial, a period which may be extended if the candidate desires a longer time in which to make up her mind or if the Matron be doubtful about her vocation. On acceptance the new probationer is placed for three months alternately in medical and surgical wards. She then obtains a fortnight's holiday, a plan which must commend itself to all who have gauged the strain of a first introduction to hospital life. On her return from this holiday she passes to three months' night duty, and ends her first year on day duty in a fourth ward. During this first year probationers are taught the elements of anatomy and physiology, with practical nursing, and bandaging under the supervision of the Matron and by the sisters in the course of the ordinary work.

### Lady Examiner from Outside.

The thoroughness of this elementary instruction is tested by an examination at the end of the first year under an experienced sister trained in another hospital. At present the lady examiner at St. George's is Miss Sleigh, formerly sister for many years at St. Bartholomew's. It is impossible to exaggerate the value of such a system to the whole nursing staff, calculated as it is to expose instantly any weakness in the practical teaching work in any part of the training school. The examination consists of a written paper, together with *viva voce* and practical tests by the lady examiner; the candidates are further examined *viva voce* by one of the surgeons and by one of the physicians of the hospital on elementary anatomy and physiology. No candidate is permitted to continue in the service of the hospital who does not pass this examination, which is of a nature to discourage any attempt at cramming and act as a sincere test of the probationer's quality no less than of the teaching organisation.

### Second and Third Years.

The practical instruction continues on the same lines as before. The classes and demonstrations deal with more advanced subjects of nursing. Nurses receive medical, surgical, and gynæcological lectures from members of the senior visiting staff and demonstrations on splints and instruments and the nursing of eye cases from the sisters. During the second year nurses receive instruction in sick-room cookery, and are examined on this subject by an examiner recognised by the Local Government Board. Except for this examination, which takes place generally during their second year, the probationers have no further anxieties until their final examination at the close of the third year. This is conducted as regards practical nursing subjects by the same outside lady examiner. The paper work is set by a physician and surgeon on the hospital staff. The second year nurses are known as staff probationers, and in the third year they may become acting staff nurses as required or according to their ability. On passing their final examination they become entitled to the certificate of training, but are required by the terms of engagement to serve the hospital a fourth year before receiving it.

### The Fourth Year.

It is in the fourth year that nurses get the opportunity of proving their fitness for responsibility. They are then staff nurses, with control of their ward during the sister's absence. To some who have given evidence of administrative talent the opportunity offers of becoming assistant housekeeper or assistant night superintendent for a period of six months, or of taking sister's holiday duty. Under certain conditions nurses are released from the fourth year of service in the hospital in order that they may become attached to the Trained Nurses' Institute.

### Theatre Work.

Practically every nurse gets a minimum of from eight to ten weeks' experience of theatre work during some period of her training subsequent to her first year. The few exceptions are women who cannot stand it, even though for ordinary work they may display genuine nursing quality. There are two general theatres at St. George's in addition to one for gynæcological work and those in the out-patients' department. In addition to the theatre sisters a staff nurse and four staff probationers are continually employed in these, and thus the Matron is able to pass the whole staff during their training through some one or other of the theatres.

### The Nurses' Quarters.

Long ago St. George's outgrew its accommodation for the nursing staff. Built in days when the nurses came in with the morning milk, St. George's faced the necessity over twenty years ago of erecting suitable premises for a Nurses' Home. It was not easy to find any site within reach of the hospital at a reasonable cost. The present Nurses'

Home is situated close to Harrod's Stores, ten minutes' quick walking from the hospital. It has eighty bedrooms and commodious sitting-rooms, with good offices. Part of the nursing staff is housed here and part in the hospital. They sleep in the hospital during their first year, except when on night duty, and at the end of this year they are passed on to the Nurses' Home. All night nurses sleep away from the hospital, and their quarters are carefully arranged in a quiet situation. All the nurses, however, have dinner and tea at the hospital, though such as sleep at the home have their breakfast and supper there. Every nurse, whether in the hospital or Nurses' Home, has a separate bedroom.

### Efficient Housekeeping.

The arrangement we have described would be held in nearly all institutions to justify entire ignorance of the cost of boarding the nurses. In addition to the problems involved in boarding nurses in two different buildings, the authorities at St. George's are faced with the necessity for preparing the food for the entire hospital, including medical and nursing staff as well as patients, in one kitchen. How, then, can it be possible, the ordinary manager will inquire, to keep any check on the expenditure for each branch of this complicated household? It is no part of the design of this article to explain the admirable system under which the steward's and secretary's department is managed in this institution. It must suffice to say that every item of expenditure is credited to its own department, and that the cost of the nurses' board is worked out week by week, presenting, with slight variations, the satisfactory average of a fraction under seven shillings per head. The Matron personally supervises the commissariat arrangements, the menu is varied, and the food of excellent quality. The kitchen premises are modern, and the kitchen staff is keenly interested in the work, an indication which is of great importance in estimating the general efficiency of the department.

### The Nurses' Sick Room.

In spite of the scarcity of space at St. George's, a really beautiful room has been reserved at the top of the building for nurses who would otherwise be exposed to the trial of being "warded." Here they can rest and recover when sent off duty for any reason, and from the raised platform near the wide windows can watch the panorama of London life spread out below them, with the leafy background of the Park, while the noises from the streets come up strangely softened to this height above.

### The Nurses' Pay and Prospects.

During their first year probationers receive £10, rising in subsequent years to £14, £22, and £26. The sisters' salary begins at £35, and rises by increments of £5 every two years to £50. The Trained Nurses' Institute affords a very fine opening for such as are suited for private work. The rate of pay in this institute, the accounts of which are in no way connected with those of the hospital, is as follows:—

First year (fourth year in service of hospital), £26 plus 10 per cent. on earnings.

Second year, £30 plus 15 per cent. on earnings.

Third year, £30 plus 24 per cent. on earnings.

Fourth year, £35 plus 30 per cent. on earnings.

Under this scheme nurses earn a very good salary from the beginning. It has been passed, moreover, by the committee that nurses who have served for two years on the staff may apply to work on the co-operative system. It is undoubtedly far more satisfactory that nurses should receive their own earnings, although the system under which they receive a fixed salary and are housed and boarded when not on duty has many important advantages, especially to women starting private nursing for the first time in London. The combination system as practised at St. George's appears to offer great safeguards to the nurses, while protecting to the full their financial interests.

### Hours on Duty and Recreations.

Probationers have at least two hours daily off duty, with four hours on Sunday. Once a month they have one whole day. The neighbourhood of the Park opens up plenty of pleasant recreation, and in the summer months they belong, if they wish, to a tennis club. In the winter they have musical evenings. There is a strong feeling of *esprit de corps* among them, and, in general, it may be said of the St. George's nurses that the lot has fallen unto them in a fair place.

### Numbers of Nursing Staff.

St. George's numbers 120 staff nurses and probationers, with twenty-five sisters; ten nurses are employed mainly outside the wards in the out-patient and light departments. Deducting these, the average number of occupied beds to each nurse on night and day staffs works out at 2.3. From twenty-six to thirty nurses complete their course and receive their certificates every year. There are no paying pupils.

The St. George's nurses get their full share of promotion after leaving their *alma mater*.

[The first article, on St. Bartholomew's Hospital, appeared on May 16.]

